# Structure of a cholinergic cell membrane

**DOI:** 10.1073/pnas.2207641119

**Published:** 2022-08-15

**Authors:** Nigel Unwin

**Affiliations:** ^a^Medical Research Council Laboratory of Molecular Biology, CB2 0QH, Cambridge, United Kingdom

**Keywords:** cholesterol, phospholipid, lipid bilayer, acetylcholine receptor, cryo-EM

## Abstract

Cell membranes are complex assemblies of proteins and lipids making transient or long-term associations that have yet to be characterized at a molecular level. Here, cryo-electron microscopy is applied to determine how phospholipids and cholesterol arrange between neighboring proteins (nicotinic acetylcholine receptors) of *Torpedo* cholinergic membrane. The lipids exhibit distinct properties in the two leaflets of the bilayer, influenced by the protein surfaces and by differences in cholesterol concentration. In the outer leaflet, the lipids show no consistent motif away from the protein surfaces, in keeping with their assumed fluidity. In the inner leaflet, where the cholesterol concentration is higher, the lipids organize into extensive close-packed linear arrays. These arrays are built from the sterol groups of cholesterol and the initial saturated portions of the phospholipid hydrocarbon chains. Together, they create an ordered ∼7 Å-thick “skin” within the hydrophobic core of the bilayer. The packing of lipids in the arrays appears to bear a close relationship to the linear cholesterol arrays that form crystalline monolayers at the air-water interface.

## Significance

While it has long been established that cell membranes are complex assemblies of proteins and bilayer-forming lipids, the inherent mobility and wide-ranging heterogeneity of the lipids have limited our ability to understand cell-membrane structure at a molecular level. Consequently, little is yet known about the protein-lipid and lipid-lipid interplay that exists in situ. The present study exploits the regular architecture of a cholinergic cell membrane to determine how the phospholipid and cholesterol organization is influenced by the protein surfaces and by differences in cholesterol concentration between the two leaflets. Lipids in the leaflet containing the most cholesterol form an ordered sterol-hydrocarbon “skin.” This hitherto unobserved hydrophobic-core structure has far-reaching implications in terms of how cholesterol-rich membranes are constructed and function.

Cell membranes are fundamental components of all living organisms, and intense efforts have been made to understand how the constituent proteins and lipids build their complex bilayer structures (1, 2). While biochemical, biophysical, and X-ray–scattering studies (3–7) have elucidated some important underlying principles, the inherent mobility and wide-ranging heterogeneity of the lipids have limited our ability to determine their three-dimensional structures at a molecular level. Most membrane-directed research has therefore focused on extracted proteins (8), or proteoliposomes (9, 10), viewing lipid molecules immobilized against the protein surfaces or trapped within a protein complex. However, these approaches do not fully recapitulate, or inform on, the protein-lipid and lipid-lipid interplay that exists in cell membranes in situ.

**Fig. 1.**
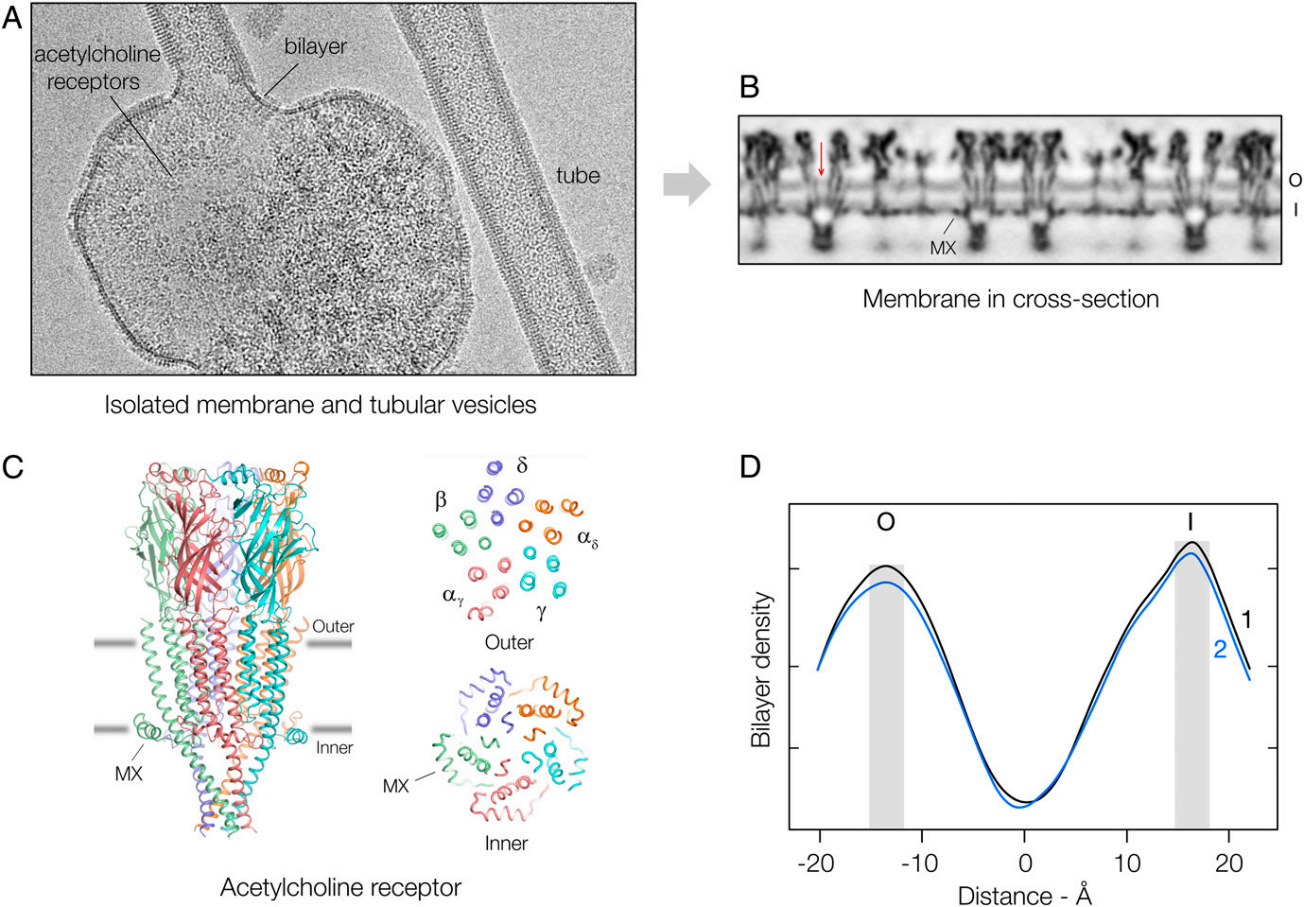
Overview and density profile of the lipid bilayer. (*A*) Isolated cell membrane and budding acetylcholine receptor tubes. In intact tissue, the receptors typically form dimer ribbons packed tightly side to side (14), a regular arrangement that is lost during membrane extraction, but is restored in the budding tubes (15). Since the protein organizes the same way in both contexts, the tube membranes may recapitulate precisely the region of the cell membrane at the synapse where receptors are most densely packed (16). (*B*) Cross-section determined from segments of tubes having the same lattice dimensions and curvature. The phospholipid headgroup regions in the outer (O) and inner (I) leaflets give rise to a pair of parallel bands in the spaces between individual receptors. MX identifies a helix of the receptor (see *C*), which substitutes for some of the phospholipid headgroups (17); the arrow points to the receptor’s central water-filled pore. (*C*) Structure of receptor and cross-sectional 12 Å-thick slabs at the levels of the gray lines, which identify the peaks of density associated with the phospholipid headgroups (17). The receptor is a heteropentamer (stoichiometry: α_δ_, α_γ_, β, γ, δ), which includes four TM helices (M1-M4) and a transverse submembrane helix, MX, in each subunit. (*D*) Mean lipid densities in maps 1 and 2 at successive radii across the lipid bilayer. The shaded columns mark the positions of the two density peaks, which are at different distances from the central low-density trough.

The present cryo-electron microscopy (cryo-EM) study exploits the regularity and high protein content of the postsynaptic cell membrane of *Torpedo* to determine its three-dimensional protein-lipid structure. This membrane has a relatively simple composition, most densely populated by a single protein (nicotinic acetylcholine receptor), embedded in a cholesterol-rich phospholipid bilayer (11–13). Moreover in tubular vesicles, which bud from the isolated membranes (Fig. 1*A*), the protein arranges on a regular but slightly varying surface lattice (15), as it does in vivo at the *Torpedo* synapse (14) and at the neuromuscular junction (18). Density maps obtained from such vesicles have shown that cholesterol attaches to specific sites on the protein in both leaflets of the bilayer and assembles into protein-bridging aggregates or microdomains (17, 19).

In earlier studies, the density maps were obtained from the tubes by helical reconstruction, using local averaging to combine the three-dimensional data from multiple subsets (17, 20). Here, the analysis is confined to single well-populated subsets, built from tube segments having the same lattice dimensions and curvature. Although requiring a much larger initial dataset, this approach succeeds in resolving longer-range details of the lipids. Thus, in the inner leaflet of the bilayer, which has the highest cholesterol content, the lipids are now seen to organize into close-packed linear arrays, building within the hydrophobic core a static sterol-hydrocarbon “skin.” Monolayer reconstitution experiments suggest that packing of lipids in these arrays bears a close relationship to the linear cholesterol arrays which form two-dimensional crystals at the air-water interface.

## Results

### Protein, Lipids, and Bilayer Density Profile.

The tubular vesicles analyzed were from a single *Torpedo* ray. Two density maps, 1 and 2, having slightly different surface lattices but the same tube curvature, were obtained by three-dimensional classification (*Methods* and *SI Appendix*, Fig. S1). Both maps were at 5.5 Å resolution (*SI Appendix*, Fig. S2) and showed lipids with good definition. A structure of the transmembrane domain of the acetylcholine receptor was also obtained by fitting an atomic model of the detergent-solubilized protein (21) to the combined 5.2 Å densities (*SI Appendix*, Figs. S2*B* and S3). This membrane-intact structure enabled quantitative estimation of the protein-excluding area available to the lipids at different levels across the bilayer (*SI Appendix*, Fig. S4).

Fig. 1*B* shows a cross-section normal to the membrane plane from map 2. The view is dominated by irregular blocks of high density, corresponding to slices through individual receptors, and by paired tracks of smoother, slightly lower density associated with the strongly electron-scattering phospholipid headgroup regions comprising the outer and inner leaflets of the lipid bilayer. Also visible, at slightly lower density still, are water in the pore of the receptor (arrow) and patches against some of the protein surfaces created by cholesterol (19), which exposes only a hydroxyl in the polar headgroup region. Finally, there is the near-central disordered hydrophobic core of the bilayer, which is the least dense region of all.

The structure of the receptor and its previously established registration with the phospholipid headgroups (17) are depicted in Fig. 1*C* (*SI Appendix*, Fig. S3). The transmembrane (TM) α-helices extend slightly beyond the lipid bilayer on the outer (extracellular) side, and the receptor is framed by a ring of submembrane α-helices, MX, which partially penetrate the bilayer on the inner side. These amphipathic helices reduce the area of surface available to the phospholipid headgroups (*SI Appendix*, Fig. S4), but alleviate the increase in free volume potentially available to their hydrocarbon chains by providing room for cholesterol within the underlying hydrophobic core (17).

The density profile of the bilayer component of this membrane (Fig. 1*D* and *SI Appendix*, Fig. S5) is similar to the bilobed profiles obtained by X-ray scattering from other cholesterol-rich membranes (e.g., 6, 7). However, the sharper appearance of the inner-leaflet peak and its 3 Å more distant location from the dividing low-density trough give rise to a notable asymmetry. The outward displacement of the peak reflects a thickening of the inner relative to the outer leaflet. This would normally be interpreted as a consequence of more orderly packing of the hydrocarbon chains imposed by the rigid sterol group: a well-documented effect of cholesterol when present in high concentration (5, 6, 22, 23). Indeed, the density maps confirm the existence of order in the inner leaflet (see next section), although not involving lipids in a fluid setting.

### Distinct Lipid Organization in the Two Leaflets.

**Fig. 2.**
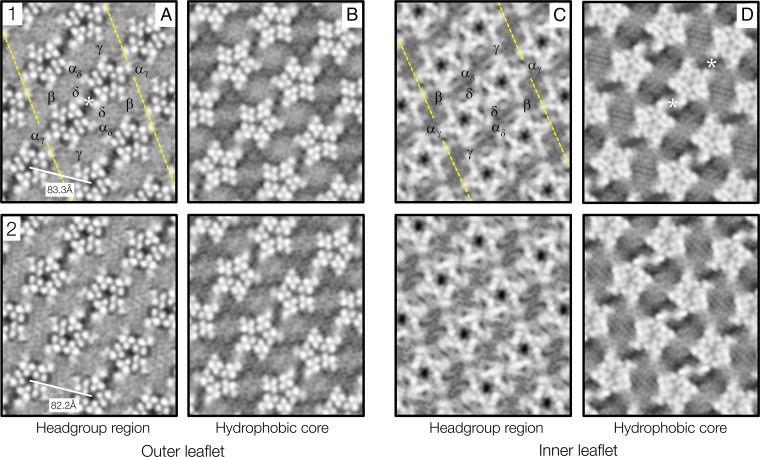
Lipid-protein organization in the outer and inner leaflets of maps 1 and 2. (*A*) and (*C*) are at levels corresponding to the peaks of density in Fig. 1*D*; (*B*) and (*D*) are 7 Å further into the interior of the bilayer. The broken yellow lines identify the well-documented ribbon of δ subunit-linked receptor dimers (14, 15, 18, 24), one of which is labeled in each leaflet to indicate the subunit arrangement. The receptors are ∼1 Å further apart along the direction of the tube axis (horizontal) in map 1 than in map 2. The asterisks mark the locations of previously described cholesterol microdomains (17). Inverted contrast.

To display the features responsible for the profile in Fig. 1*D*, the three-dimensional maps were sampled in cylindrical shells at various tube radii and presented as planar sections, each representing a particular bilayer level, or depth. Fig. 2 shows sections from both maps at the level of the high-density peaks and at 7 Å further into the bilayer interior. According to the interpretation of X-ray–scattering measurements (e.g., 6, 7), the high-density peaks identify the approximate locations of the phosphate moieties of the phospholipid headgroups, whereas the 7 Å level corresponds to a portion of the hydrophobic core harboring the sterol groups of cholesterol and the initial saturated portions of the phospholipid hydrocarbon chains.

In the outer leaflet, at the level of the density peak (Fig. 2*A*), the section cuts through the TM helices near their extracellular ends, with the large phospholipid headgroups occupying most of the remaining area. Exceptions are the water-filled patches lying between the δ subunits of neighboring receptors and smaller patches next to specific protein surfaces, signifying the presence of aggregated or immobilized cholesterol in the underlying leaflet (19). There is no evidence for similar specific interactions by the phospholipids, suggesting that their associations with the protein are more transient (although there may be exceptions (21, 25)). Unsurprisingly, in view of their heterogeneity and consequential disorder, the headgroups in general are not resolved and so have a rather uniform appearance.

At the 7 Å level (Fig. 2*B*), the sterol groups and hydrocarbon chains also give rise to a fairly uniform appearance, as would be expected, given their likely mobile nature and their similar densities. In fact, molecular dynamics simulations of cholesterol-rich membranes show that the rigid sterol groups, with protruding methyls, do not pack tightly as do the all-*trans* hydrocarbon chains (22). Therefore, the slightly lower densities underlying the water-filled patches in Fig. 2*A*, and in regions further from the protein surfaces, probably both arise from elevated concentrations of cholesterol.

At the density peak of the inner leaflet (Fig. 2*C*), the section cuts through the MX helices, which restrict the area available to the phospholipid headgroups to ∼25% of the total membrane area (compared with ∼50% in the outer leaflet; *SI Appendix*, Fig. S4). Again, the headgroups appear rather featureless. However, further into the interior of the bilayer (Fig. 2*D*), and in marked contrast to their appearance in Fig. 2*B*, the sterol groups and hydrocarbon chains form organized assemblies. These extend far from the protein surfaces and the cholesterol aggregates directly underlying the MX helices (asterisks, Fig. 2*D*) (17) and consist predominantly of close-packed linear arrays.

The sterol-hydrocarbon arrays have several defining properties. First, they are static molecular assemblies, since the same array-motifs occur in maps calculated independently from half-datasets (*SI Appendix*, Fig. S6*A*). Second, their organization—different in maps 1 and 2—is evidently determined by the spaces made available by the different lattices, not directly by the protein surfaces. Third, they are visible within the hydrophobic core over a thickness of about 7 Å (*SI Appendix*, Fig. S6*B*) and hence span nearly the entire sterol-occupied portion of the bilayer.

### Cholesterol Monolayer Films.

**Fig. 3.**
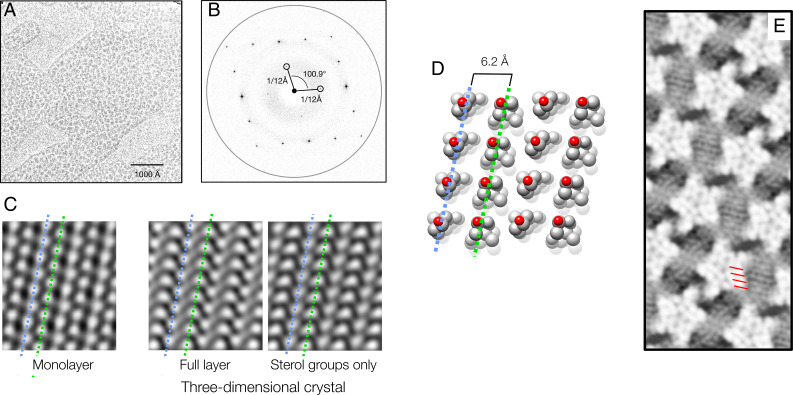
Linear arrays of cholesterol and phospholipids. (*A*) Image of a cholesterol monolayer film (blotchy areas) on a thin carbon support. (*B*) Fourier transform showing diffraction spots and contrast transfer function-modulated background intensities; the ring is at a resolution of 3.6 Å. (*C*) Filtered image of the monolayer film (*Left*) and projection structures calculated from the closely related triclinic crystal layer (*Right*): complete layer and with the isooctyl chains removed. (*D*) Packing of cholesterol in the triclinic crystal layer (26): the molecules align with slightly different tilts along rows, wherein they face alternately in opposite directions. Equivalent rows in *C* and *D* are identified by blue and green dashed lines. (*E*) Detail from Fig. 2*D*, showing close matching of the 6.2 Å row periodicities (red bars) and the periodicities present in the cell membrane.

To shed further light on the molecular nature of these arrays, I examined the organization of pure cholesterol in monolayer films created at the air-water interface (*Methods*), using cryo-EM. Cholesterol in these films (Fig. 3*A*) was found to crystallize on a near-identical lattice (*a* = 12.01 ± 0.03 SD Å, *b* = 12.07 ± 0.06 SD Å, γ = 100.9 ± 0.12° SD; Fig. 3*B*) to that of the planar layers comprising the three-dimensional triclinic crystal (*a* = 12.39 Å, *b* = 12.41 Å, γ = 100.8°) (26), a polymorphic form present in atherosclerotic plaques (26, 27). However, the flexible isooctyl chains appeared to clump in small patches (Fig. 3*A*), a feature corroborated by Fourier-filtered images (Fig. 3*C*), which resemble most closely the projection structure calculated from the three-dimensional crystal layer (Fig. 3*D*) with the isooctyl chains removed. Thus, in the monolayer film, the ordering is retained only in the sterol ring portion of the molecule, not throughout. Electron diffraction of the monolayer films at room temperature yields the same “diamond” spot pattern as in Fig. 3*B* (*SI Appendix*, Fig. S7), confirming that the ordering is not cryo-induced. Most intriguingly, in the context of the cell membrane, the crystal lattice is built from linear arrays of cholesterol having a periodicity (6.0–6.2 Å), which is very similar to that of the observed sterol-hydrocarbon arrays in regions where they pack side by side (Fig. 3*E*).

## Discussion

Cholesterol in *Torpedo* and other cholinergic membranes is required to enable the classical physiological transitions associated with rapid switching of receptors between closed (or resting), open, and desensitized states (28, 29). Previous cryo-EM studies have investigated how it organizes next to the protein surfaces, illuminating its role in stabilizing the transmembrane architecture of the receptor (17) and suggesting how, by bridging neighboring receptors, it facilitates cooperative interactions (30). The current analysis looks at how cholesterol, as well as the other bilayer lipids, organize in regions distant from the protein surfaces. Accordingly, it provides additional, much sought-after insight into how cell membranes in general are constructed, particularly in locations where protein and cholesterol are both present in high concentration (often referred to as lipid rafts (31)).

The concentration of cholesterol is 40–46 mol % of the total lipids in acetylcholine receptor-rich *Torpedo* membranes (11–13), but how it is partitioned between the two leaflets is unknown. This is difficult to estimate directly from the density maps, which cannot distinguish 1:1 cholesterol-phospholipid complexes (23) in which the two kinds of lipid may have tight headgroup interactions (32) and because of “blurring” arising from mobility of the lipids. However, the protein-bridging patches identified previously as cholesterol microdomains (asterisks, Fig. 2) (17, 19) occupy about twice the total area in the inner leaflet that they occupy in the outer leaflet, suggesting that concentrations in the two leaflets may differ by roughly this factor. Such a disparity would be in line with the asymmetric nature of the bilayer density profile (Fig. 1*D*). It also has precedence in myelin membrane, which exhibits a similar asymmetric profile and has an estimated two-times difference in cholesterol concentration between leaflets (6). Given the high overall percentage figure, the cholesterol concentration in the inner leaflet must therefore be well over 50 mol %, the saturating amount measured in lecithin bilayers (33).

**Fig. 4.**
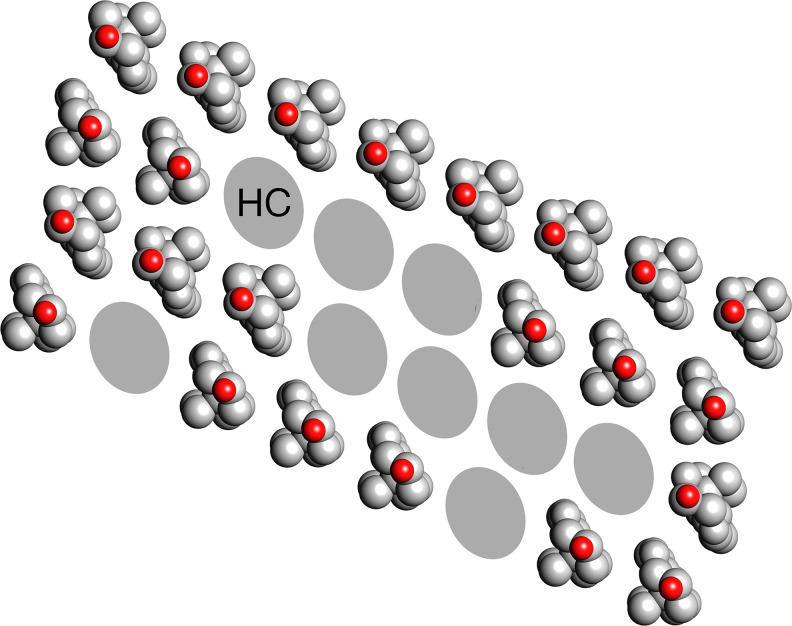
Ordering of the hydrocarbon chains imposed by cholesterol. It is proposed that the cholesterol monomers, no longer stable under saturating conditions in a fluid hydrocarbon environment, self-assemble in linear arrays as in the monolayer films. Side-to-side interactions also occur involving not only other sterol groups but also, in a random way, the paired hydrocarbon chains (HC) of the phospholipids. Because the density maps average over many different sterol/hydrocarbon combinations, continuous lines appear, which are roughly the same distance apart as are the linear arrays of cholesterol in the monolayer films (Fig. 3*E*).

Under saturating conditions, the cholesterol monomers would be expected to form stable associations and could plausibly polymerize, i.e., self-assemble in linear arrays, as in the monolayer films. Fig. 4 shows how the sterol-hydrocarbon motifs might then develop, by sideways expansion and incorporation of the hydrocarbon chains, into close-packed networks like those in Fig. 2*D*. There is no evidence for any phase separation between the sterol and hydrocarbon components within the highly confined protein-walled spaces. Moreover, 1:1 associations of phospholipids with cholesterol can be accommodated because the two kinds of lipid have near-equal cross-sections when present in near-equal amounts (22). The observed thickness and reproducibility of the motifs (*SI Appendix*, Fig. S6), their distinct arrangements to fit the differing small volumes available, and their near 6.2 Å periodicity all point to a cholesterol assembly-driven origin.

Hence, while in the outer leaflet, the lipids are presumably in a fluid state like that of a liquid crystalline phase studied in model membrane vesicles (34); in the inner leaflet, they would be constrained in their lateral motion as a result of the cholesterol polymerization. Apparently, the thin sterol-occupied portion of the inner leaflet forms a static layer, or skin, imparting the membrane with physical properties that would be different from other regions where cholesterol is more dilute. There remains the possibility that the observed lipid ordering is not normally present in the tube membrane but has been induced artificially by the plunge-freezing. However, this seems unlikely in view of the room-temperature stability of ordered sterol groups in the monolayer films. Also, rapid phase transitions, like those studied in simplified bilayer systems, generally do not occur in biological membranes (2).

Of wider significance, the sterol-hydrocarbon layer incorporates only the initial saturated portions of the hydrocarbon chains, which are the same for all common phospholipids. The invariably heterogeneous headgroup and tail portions are not involved. The ordered hydrophobic-core structure described here might therefore be present in protein- and cholesterol-rich cell membranes wherever cholesterol in one of the leaflets exceeds saturating amounts. This could be a common occurrence, since cholesterol typically constitutes 30–40 mol % of the total lipids in animal cell membranes (35), and many such membranes contain specialized regions (e.g., lipid rafts), where cholesterol is further enriched.

## Methods

### Specimen Preparation.

Acetylcholine receptor-rich membranes were isolated from fresh *T. marmorata* electric organ (15). Tubular vesicles, reconstituting the physiological protein organization, were obtained by incubating the membranes in low-salt buffer (100 *mM* sodium cacodylate, 1 *mM* calcium chloride, pH 7) at 17 °C for 2 wk.

Cholesterol monolayer films were produced at the air-water interface, using 60 μL (4 mm diameter) water-filled Teflon wells. A total of 5 μL of chloroform containing 1 mg/mL high-purity cholesterol (Sigma) was injected into the bottom of the well. The mixture was incubated in a humid atmosphere at 4 °C for 24 h, allowing the chloroform to evaporate and the cholesterol monolayer to develop. A thin hydrophobic carbon film, supported by a perforated EM grid (Quantifoil R 0.6/1) was placed on the water surface and then carefully withdrawn, bringing the cholesterol monolayer with it. The grids were stored in liquid nitrogen before examination by cryo-EM or used directly in room-temperature experiments.

### Cryo-EM and Image Processing.

Aliquots of the tube-containing solution were applied to perforated EM grids and blotted to retain the specimens in a thin aqueous film, before plunging into liquid nitrogen-cooled ethane. Micrographs of straight ice-embedded tubes were recorded at 300 k*V* on a FEI Titan Krios electron microscope (Thermo Fisher Scientific), using a Falcon 3 direct-electron detector operating in integrating mode. Underfocus values ranged from 1 to 2.8 μ*m*. The calibrated pixel size on the specimen was 1.34 Å. The total dose was 40 e Å^−2^, fractionated from 79 frames. Micrograph frame stacks were drift corrected and dose weighted using MotionCor2 (36). Local contrast transfer functions (*CTF*s) were estimated from the aligned, nondose-weighted micrographs using Gctf (37). All subsequent image processing steps were performed in RELION (38, 39). Drift-correction and dose-weighting were applied similarly to the cholesterol monolayer films, and filtered images were obtained from the *CTF*-corrected Fourier transforms by masking out all areas except for small circular windows around each of the diffraction spots.

Analysis of the tubular vesicles (*SI Appendix*, Fig. S1) was based solely on the (−17, 5) helical family (40), the family most commonly encountered of many in the preparation. In brief, three rounds of two-dimensional classification, applied to segments extracted from 1,993 images, yielded ∼85% of sufficient quality for further processing. Three-dimensional classification was conducted in two rounds, first, to separate out segments having the same lattice dimensions and curvature (i.e., helical twist, rise, and tube radius), and second, to separate out segments having matching transmembrane structures. An annular mask was applied in the second round to include just the transmembrane portion. The final unmasked density maps were obtained from two well-populated subsets derived by this procedure (*SI Appendix*, Fig. S2). A value for the regularization parameter T = 10 was applied throughout, except in the second round, where its value was increased to 20 to ensure that details associated with the lipids contributed significantly to the refinement.

For Fig. 2, the densities at successive levels through the lipid bilayer were calculated from the three-dimensional volumes in cylindrical shells around the tube axes and displayed as planar sections. Fall-off in contrast, due mainly to inelastic scattering from the thick ice (41), was compensated for by applying a modest temperature factor (B = −120 A^2^). Fitting of the atomic model of the receptor, calculation of projected densities from the cholesterol crystals, and preparation of the structural figures were done using DireX (42), UCSF Chimera (43) and PyMOL (44). 

## Supplementary Material

Supplementary File

## Data Availability

The cryo-EM density maps were deposited in the Electron Microscopy Data Bank with the following accession codes: map 1 (EMD-14942) (45) and map 2 (EMD-14946) (46).

## References

[1] S. J. Singer, G. L. Nicolson, The fluid mosaic model of the structure of cell membranes. Science 175, 720–731 (1972). 4333397 10.1126/science.175.4023.720

[2] R. B. Gennis, Biomembranes: Molecular Structure and Function (Springer-Verlag New York Inc., 1989).

[3] M. S. Bretscher, Asymmetrical lipid bilayer structure for biological membranes. Nat. New Biol. 236, 11–12 (1972). 4502419 10.1038/newbio236011a0

[4] D. Branton, Fracture faces of frozen membranes. Proc. Natl. Acad. Sci. U.S.A. 55, 1048–1056 (1966). 5334198 10.1073/pnas.55.5.1048 PMC224277

[5] Y. K. Levine, M. H. F. Wilkins, Structure of oriented lipid bilayers. Nat. New Biol. 230, 69–72 (1971). 5279040 10.1038/newbio230069a0

[6] D. L. D. Caspar, D. A. Kirschner, Myelin membrane structure at 10 A resolution. Nat. New Biol. 231, 46–52 (1971). 5283387 10.1038/newbio231046a0

[7] N. P. Franks, Structural analysis of hydrated egg lecithin and cholesterol bilayers. I. X-ray diffraction. J. Mol. Biol. 100, 345–358 (1976). 943548 10.1016/s0022-2836(76)80067-8

[8] K. R. Vinothkumar, R. Henderson, Structures of membrane proteins. Q. Rev. Biophys. 43, 65–158 (2010). 20667175 10.1017/S0033583510000041 PMC3604715

[9] T. Gonen , Lipid-protein interactions in double-layered two-dimensional AQP0 crystals. Nature 438, 633–638 (2005). 16319884 10.1038/nature04321 PMC1350984

[10] W. Qiu , Structure and activity of lipid bilayer within a membrane-protein transporter. Proc. Natl. Acad. Sci. U.S.A. 115, 12985–12990 (2018). 30509977 10.1073/pnas.1812526115 PMC6304963

[11] J.-L. Popot, R. A. Demel, A. Sobel, L. L. M. Van Deenen, J.-P. Changeux, Interaction of the acetylcholine (nicotinic) receptor protein from *Torpedo marmorata* electric organ with monolayers of pure lipids. Eur. J. Biochem. 85, 27–42 (1978). 639821 10.1111/j.1432-1033.1978.tb12209.x

[12] J. M. Gonzalez-Ros, M. Llanillo, A. Paraschos, M. Martinez-Carrion, Lipid environment of acetylcholine receptor from *Torpedo californica*. Biochemistry 21, 3467–3474 (1982). 7115681 10.1021/bi00257a033

[13] N. P. Rotstein, H. R. Arias, F. J. Barrantes, M. I. Aveldaño, Composition of lipids in elasmobranch electric organ and acetylcholine receptor membranes. J. Neurochem. 49, 1333–1340 (1987). 2822851 10.1111/j.1471-4159.1987.tb00996.x

[14] J. E. Heuser, S. R. Salpeter, Organization of acetylcholine receptors in quick-frozen, deep-etched, and rotary-replicated *Torpedo* postsynaptic membrane. J. Cell Biol. 82, 150–173 (1979). 479296 10.1083/jcb.82.1.150 PMC2110412

[15] A. Brisson, P. N. T. Unwin, Tubular crystals of acetylcholine receptor. J. Cell Biol. 99, 1202–1211 (1984). 6480689 10.1083/jcb.99.4.1202 PMC2113304

[16] N. Unwin, Nicotinic acetylcholine receptor and the structural basis of neuromuscular transmission: Insights from *Torpedo* postsynaptic membranes. Q. Rev. Biophys. 46, 283–322 (2013). 24050525 10.1017/S0033583513000061 PMC3820380

[17] N. Unwin, Protein-lipid architecture of a cholinergic postsynaptic membrane. IUCrJ 7, 852–859 (2020). 10.1107/S2052252520009446 PMC7467168 32939277

[18] N. Hirokawa, J. E. Heuser, Internal and external differentiations of the postsynaptic membrane at the neuromuscular junction. J. Neurocytol. 11, 487–510 (1982). 6980263 10.1007/BF01257990

[19] N. Unwin, Segregation of lipids near acetylcholine-receptor channels imaged by cryo-EM. IUCrJ 4, 393–399 (2017). 10.1107/S2052252517005243 PMC5571802 28875026

[20] A. Miyazawa, Y. Fujiyoshi, M. Stowell, N. Unwin, Nicotinic acetylcholine receptor at 4.6 Å resolution: transverse tunnels in the channel wall. J. Mol. Biol. 288, 765–786 (1999). 10329178 10.1006/jmbi.1999.2721

[21] M. M. Rahman , Structural mechanism of muscle nicotinic receptor desensitization and block by curare. Nat. Struct. Mol. Biol. 29, 386–394 (2022). 35301478 10.1038/s41594-022-00737-3 PMC9531584

[22] C. L. Wennberg, D. van der Spoel, J. S. Hub, Large influence of cholesterol on solute partitioning into lipid membranes. J. Am. Chem. Soc. 134, 5351–5361 (2012). 22372465 10.1021/ja211929h

[23] W.-C. Hung, M.-T. Lee, F.-Y. Chen, H. W. Huang, The condensing effect of cholesterol in lipid bilayers. Biophys. J. 92, 3960–3967 (2007). 17369407 10.1529/biophysj.106.099234 PMC1868968

[24] H. W. Chang, E. Bock, Molecular forms of acetylcholine receptor. Effects of calcium ions and a sulfhydryl reagent on the occurrence of oligomers. Biochemistry 16, 4513–4520 (1977). 410440 10.1021/bi00639a028

[25] A. Sridhar , Regulation of a pentameric ligand-gated ion channel by a semiconserved cationic lipid-binding site. J. Biol. Chem. 297, 100899 (2021). 34157288 10.1016/j.jbc.2021.100899 PMC8327344

[26] B. M. Craven, Crystal structure of cholesterol monohydrate. Nature 260, 727–729 (1976). 1264248 10.1038/260727a0

[27] N. Varsano, I. Fargion, S. G. Wolf, L. Leiserowitz, L. Addadi, Formation of 3D cholesterol crystals from 2D nucleation sites in lipid bilayer membranes: Implications for atherosclerosis. J. Am. Chem. Soc. 137, 1601–1607 (2015). 25584426 10.1021/ja511642t

[28] M. Criado, H. Eibl, F. J. Barrantes, Effects of lipids on acetylcholine receptor. Essential need of cholesterol for maintenance of agonist-induced state transitions in lipid vesicles. Biochemistry 21, 3622–3629 (1982). 7115688 10.1021/bi00258a015

[29] J. E. Baenziger, J. A. Domville, J. P. D. Therien, The role of cholesterol in the activation of nicotinic acetylcholine receptors. Curr. Top. Membr. 80, 95–137 (2017). 28863823 10.1016/bs.ctm.2017.05.002

[30] N. Unwin, Protein-lipid interplay at the neuromuscular junction. Microscopy (Oxf.) 71 (suppl. 1), i66–i71 (2022). 34226930 10.1093/jmicro/dfab023 PMC8855523

[31] D. Lingwood, K. Simons, Lipid rafts as a membrane-organizing principle. Science 327, 46–50 (2010). 20044567 10.1126/science.1174621

[32] J. B. Finean, Interaction between cholesterol and phospholipid in hydrated bilayers. Chem. Phys. Lipids 54, 147–156 (1990).

[33] H. Lecuyer, D. G. Dervichian, Structure of aqueous mixtures of lecithin and cholesterol. J. Mol. Biol. 45, 39–57 (1969). 5343454 10.1016/0022-2836(69)90208-3

[34] D. A. Brown, E. London, Structure and origin of ordered lipid domains in biological membranes. J. Membr. Biol. 164, 103–114 (1998). 9662555 10.1007/s002329900397

[35] S. Munro, Lipid rafts: Elusive or illusive? Cell 115, 377–388 (2003). 14622593 10.1016/s0092-8674(03)00882-1

[36] X. Li , Electron counting and beam-induced motion correction enable near-atomic-resolution single-particle cryo-EM. Nat. Methods 10, 584–590 (2013). 23644547 10.1038/nmeth.2472 PMC3684049

[37] K. Zhang, Gctf: Real-time *CTF* determination and correction. J. Struct. Biol. 193, 1–12 (2016). 26592709 10.1016/j.jsb.2015.11.003 PMC4711343

[38] S. H. Scheres, *RELION*: Implementation of a Bayesian approach to cryo-EM structure determination. J. Struct. Biol. 180, 519–530 (2012). 23000701 10.1016/j.jsb.2012.09.006 PMC3690530

[39] S. He, S. H. W. Scheres, Helical reconstruction in *RELION*. J. Struct. Biol. 198, 163–176 (2017). 28193500 10.1016/j.jsb.2017.02.003 PMC5479445

[40] C. Toyoshima, N. Unwin, Three-dimensional structure of the acetylcholine receptor by cryoelectron microscopy and helical image reconstruction. J. Cell Biol. 111, 2623–2635 (1990). 2277076 10.1083/jcb.111.6.2623 PMC2116367

[41] J. L. Dickerson, P. H. Lu, D. Hristov, R. E. Dunin-Borkowski, C. J. Russo, Imaging biological macromolecules in thick specimens: The role of inelastic scattering in cryoEM. Ultramicroscopy 237, 113510 (2022). 35367900 10.1016/j.ultramic.2022.113510 PMC9355893

[42] G. F. Schröder, A. T. Brunger, M. Levitt, Combining efficient conformational sampling with a deformable elastic network model facilitates structure refinement at low resolution. Structure 15, 1630–1641 (2007). 18073112 10.1016/j.str.2007.09.021 PMC2213367

[43] E. F. Pettersen , UCSF Chimera--a visualization system for exploratory research and analysis. J. Comput. Chem. 25, 1605–1612 (2004). 15264254 10.1002/jcc.20084

[44] W. L. Delano, The PyMOL Graphics System (Version 2.0, Schrödinger, LLC, New York, NY, 2017).

[45] N. Unwin, Structure of a cholinergic cell membrane. Electron Microscopy Data Bank https://www.ebi.ac.uk/emdb/EMD-14942. Deposited 22 July 2022.

[46] N. Unwin, Structure of a cholinergic cell membrane. Electron Microscopy Data Bank https://www.ebi.ac.uk/emdb/EMD-14946. Deposited 22 July 2022.

