# Supplementary material for: Structure of a cholinergic cell membrane
Source: Proc Natl Acad Sci U S A. 2022 Aug 15;119(34):e2207641119. doi: 10.1073/pnas.2207641119 (PMC9407305; doi:10.1073/pnas.2207641119)
Supplement: Supplementary File [file pnas.2207641119.sapp.pdf]

## Supplementary Information for

# Structure of a Cholinergic Cell Membrane

Nigel Unwin

Corresponding Author: Nigel Unwin
Email: unwin@mrc-lmb.cam.ac.uk

**This PDF file includes:**

Figures S1 to S7
SI References

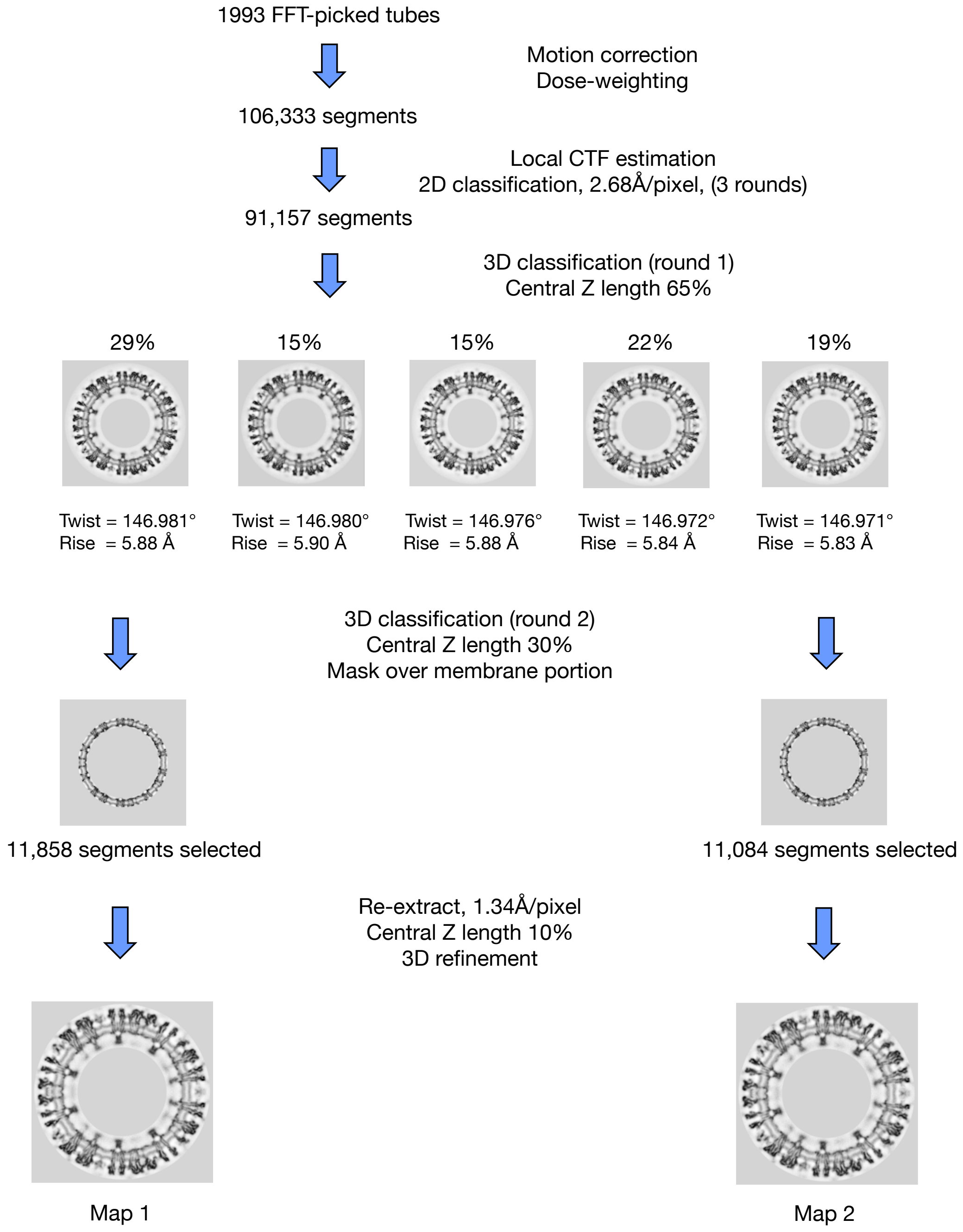


**Fig. S1. Image processing workflow.** Tubes of the (-17, 5) helical family (1) were selected by inspection of their Fourier transforms (2) and processed following procedures in *RELION* (3), which sorted segments into distinct classes according to helical twist and rise and tube radius. Segment sizes of 512 and 800 pixels were used with the 2.68 and 1.34 Å sampling, respectively. The inter-segment distance was 80 Å.

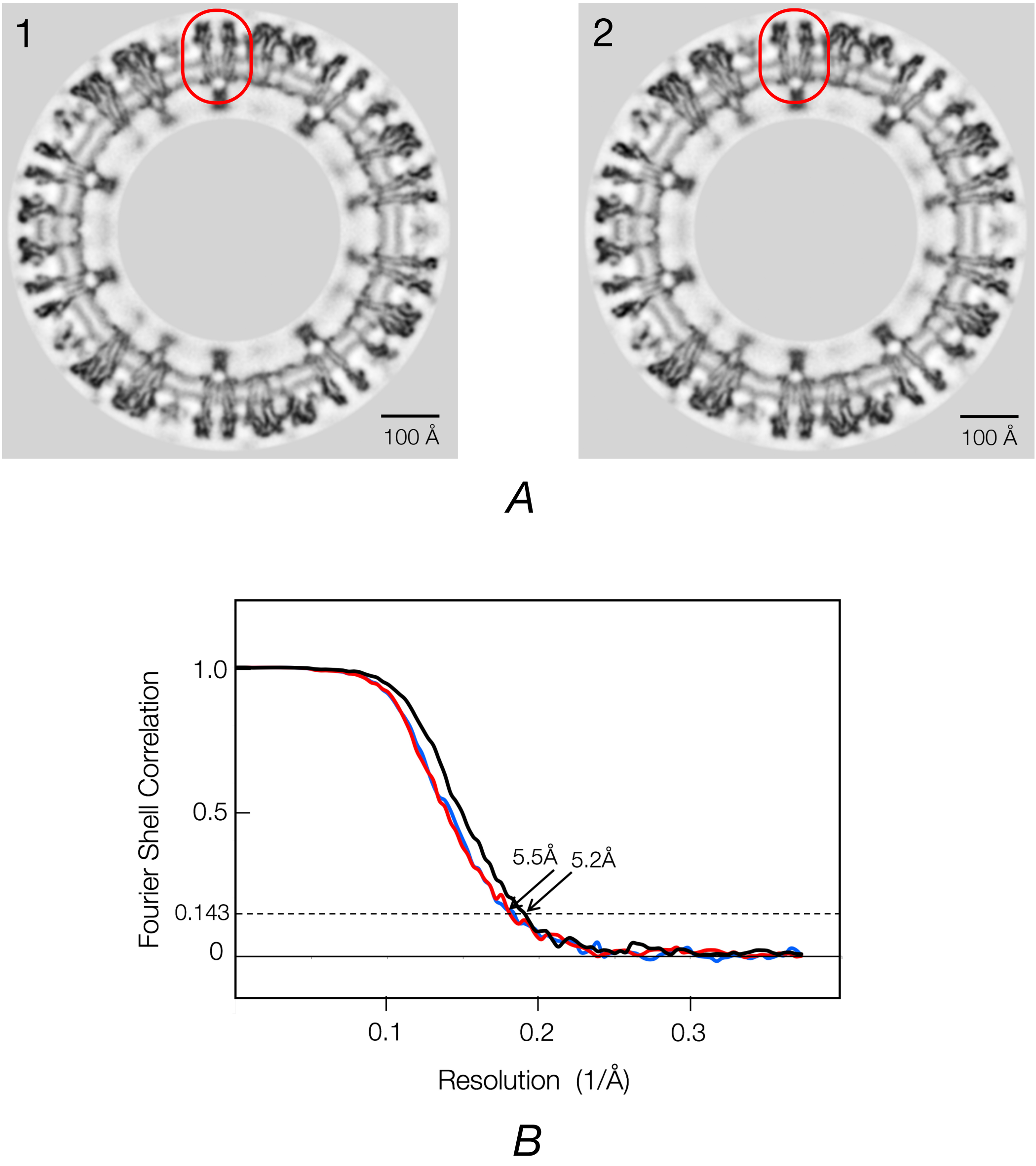


**Fig. S2. Characterisation of Maps 1 and 2.** (*A*) Central cross-sections through the two maps. The reconstructed tubes have the same diameter but different helical parameters: $twist_1$ = 146.981°, $rise_1$ = 5.88 Å; $twist_2$ = 146.971°, $rise_2$ = 5.83 Å. Red boxes indicate the volumes cut out for Fourier shell correlation (*FSC*) determinations. (*B*) *FSC* curves obtained by comparison of the independent half-datasets from 1 and 2 (red and blue curves), and by comparison of the two full datasets (black curve). The resolutions estimated by the *FSC*=0.143 criterion are 5.5 Å for the individual maps and 5.2 Å for the average.

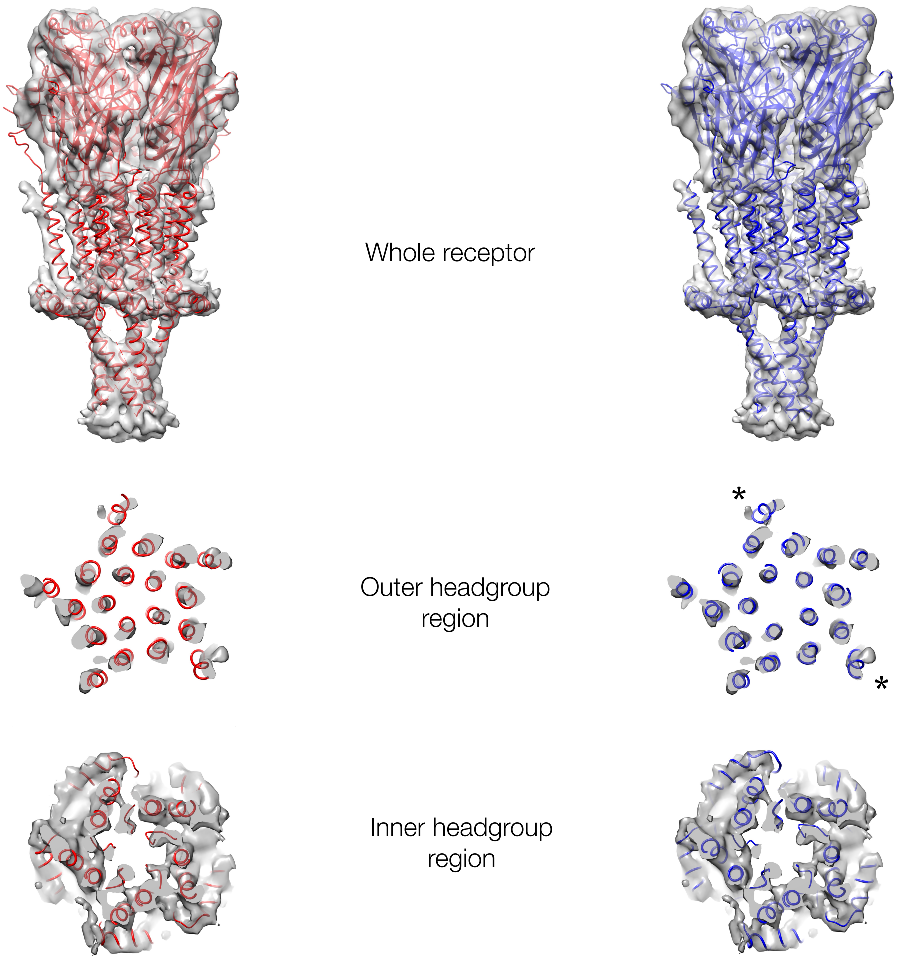


**Fig. S3. Fit of acetylcholine-receptor structure to the combined 5.2 Å density map**. The structural model is of the detergent-solubilised protein in an unliganded conformation (PDB ID code: 7SMQ) (4). The TM helices in this model, as in an earlier model (5, 6), are more tightly packed than they are in the receptor prior to its extraction from the membrane. To correct for the inward displacements of the helices, the membrane-spanning domain was refined in *DireX* (7) by maximising the correlation between the densities computed from the model and the experimental densities (sharpened, B = -400 $Å^2$) (8), while retaining the original secondary structure. The rest of the structure was left unchanged. Shown are examples of the model superimposed on the experimental densities before (left) and after (right) correction. In addition to the improved fit of the TM helices, the MX helices are more nearly co-planar, and parallel to the membrane surface, after refinement. The peripheral (M4) helices of the $\alpha$ subunits uniquely are kinked in the outer headgroup region (Fig. 1*C*) (4), and have matching elongated/bifurcated densities (asterisks), which are evident also in Fig. 2*A*. The unassigned density at the base of the receptor (also visible in Fig. 1*B*) most probably arises from the attached (but not helically ordered) protein rapsyn (9, 10).

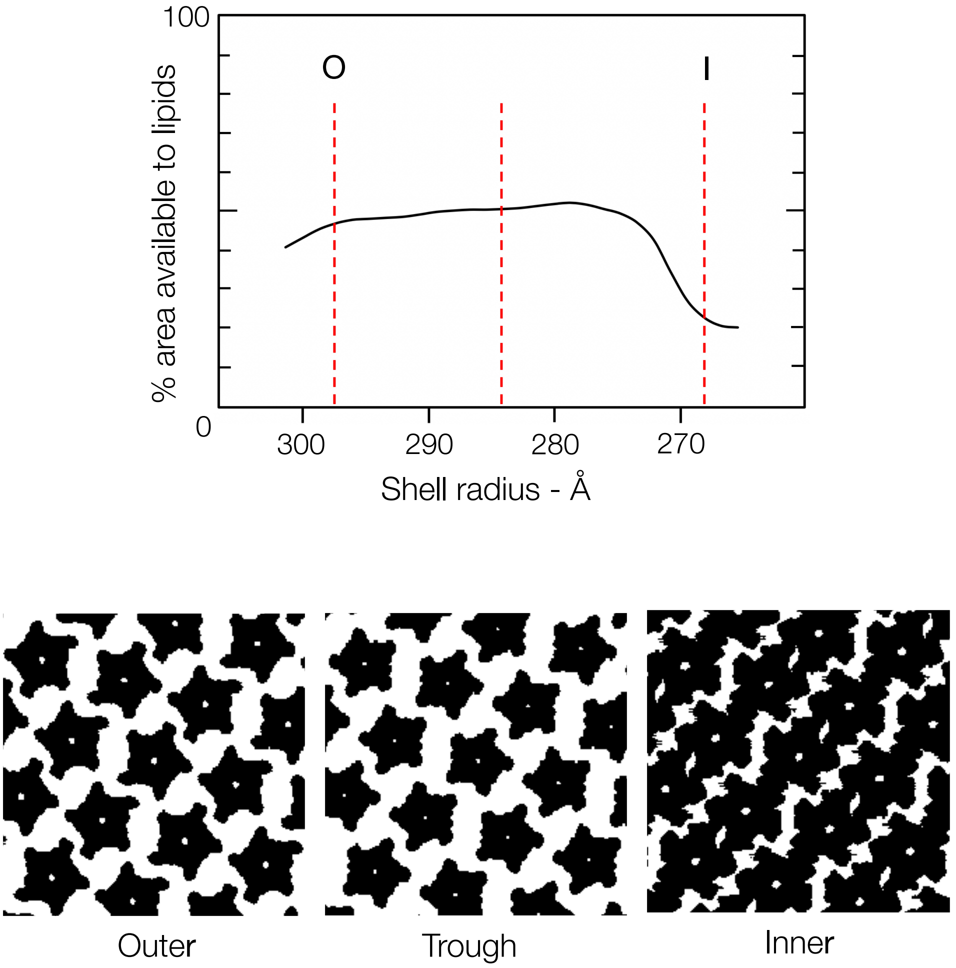


**Fig. S4. Estimation of the protein-excluding area available to the lipids at successive tube radii.** An artificial tube was constructed from the coordinates of the modelled receptor (Fig. S3), using the helical parameters defining Map 2. This 'pdb' tube was then converted to a density map at 10 Å resolution and assessed in successive cylindrical shells at 1.34 Å intervals, assuming a threshold for the protein consistent with its molecular mass. Shown is the estimated percentage of area available to the lipids, in the bilayer region, determined from the proportion of empty space present in each shell. The vertical lines at radii of 297.5 Å, 284.4 Å and 268 Å, indicate the locations of the outer-leaflet peak, low density trough and inner-leaflet peak in Fig. 1*D*. The corresponding planar sections are shown below (protein, black; empty spaces, white).

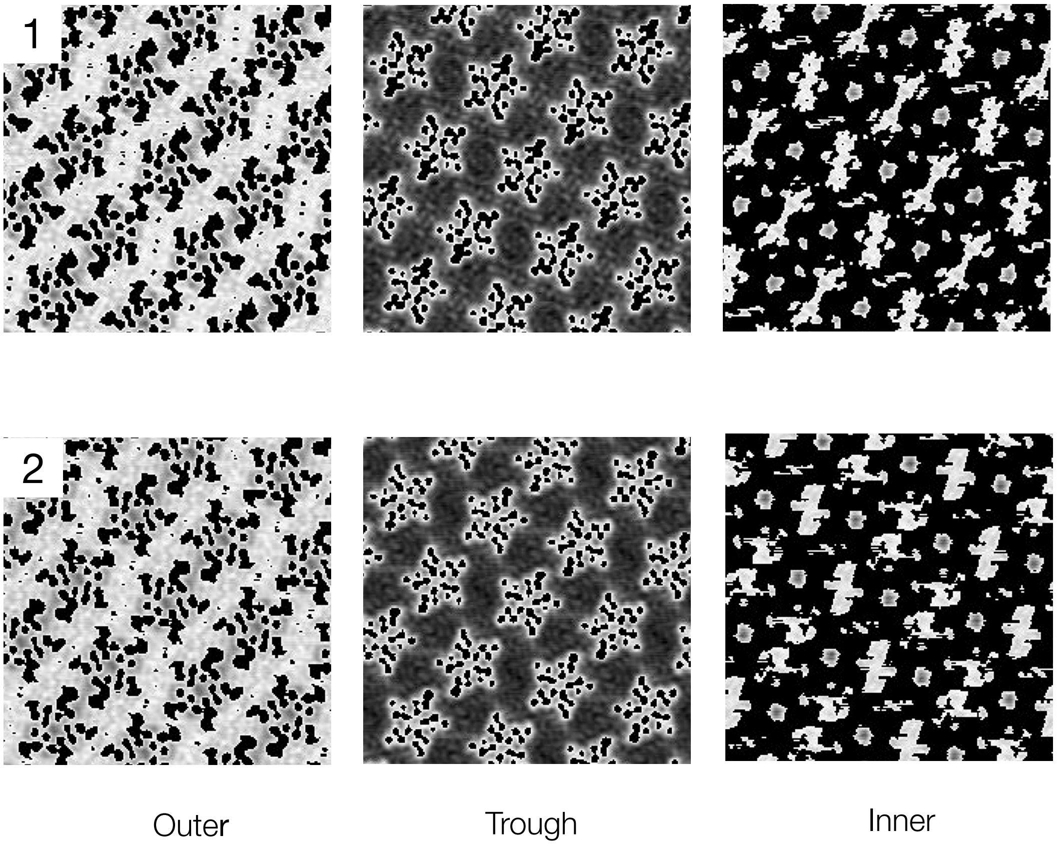


**Fig. S5. Estimation of the variation in mean lipid densities across the bilayer**. Cylindrical shells were calculated from the two maps at 1.34 Å intervals, as in Fig. S4, and a threshold was applied to block out the high densities attributable to protein. The densities in the remaining areas were then averaged. Shown are planar sections corresponding to the outer-leaflet peak (left), low-density trough (middle), and inner-leaflet peak (right) in Fig. 1*D* (blacked-out areas, protein). The shape of the bilayer profile was not significantly affected by minor variations in the threshold value (or, for example, by corrections to account for water in the pore).

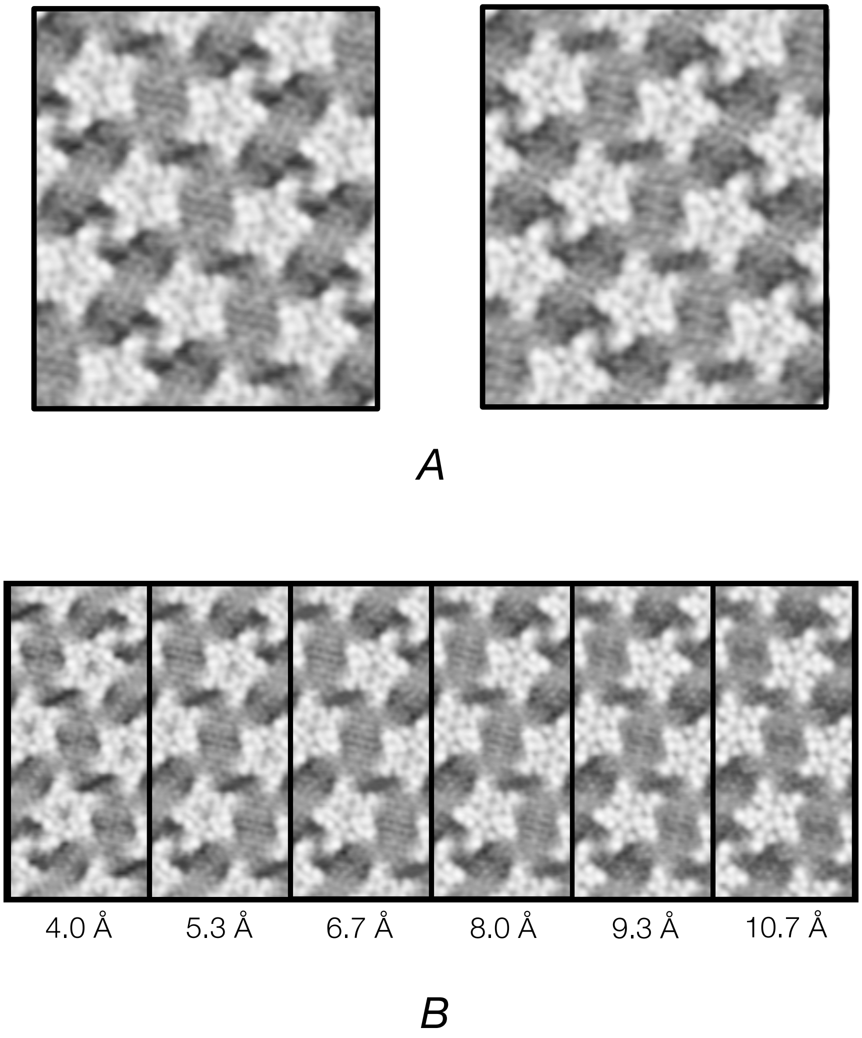


**Fig. S6**. **Characterisation of the linear sterol-hydrocarbon arrays.** (*A*) Equivalent sections through independent reconstructions made from half-datasets (Map 1), showing similar - although more noisy - lipid features in both. (Neither half-dataset includes segments from images that are included in the other half-dataset). (*B*) Sections at successive depths through the hydrophobic core of the bilayer, showing regular features spanning a total thickness of about 7 Å. The numbers correspond to distances from the density peak in the inner leaflet (I, Fig.1*D*).

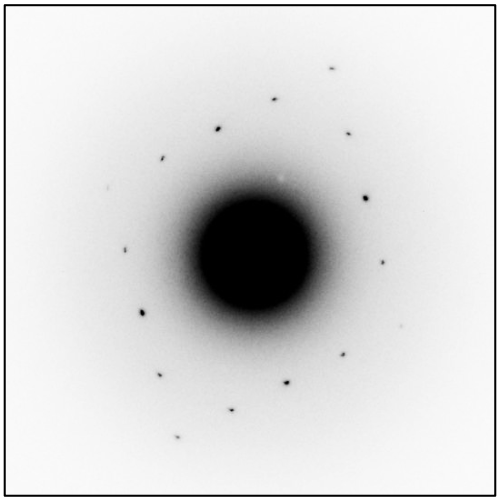

**Fig. S7. Electron diffraction pattern from a cholesterol monolayer film recorded at room temperature.** The pattern of spots is the same as that appearing in Fourier transforms of images recorded at near liquid-nitrogen temperature (Fig. 3*B*)

## References


1. C. Toyoshima, N. Unwin, Three-dimensional structure of the acetylcholine receptor by cryoelectron microscopy and helical image reconstruction *J. Cell Biol.* **111**, 2623-2635 (1990).
2. N. Unwin, Segregation of lipids near acetylcholine-receptor channels imaged by cryo-EM. *IUCrJ* **4**, 393-399 (2017).
3. S. He, S. H. W. Scheres, (2017). Helical reconstruction in *RELION*. *J. Struct. Biol.* **198**, 163-176 (2017).
4. M.M. Rahman *et al*., Structural mechanism of muscle nicotinic receptor desensitization and block by curare *Nat. Struct. Mol. Biol.* **29**, 386-394 (2022).
5. M.M. Rahman *et al*., Structure of the native muscle-type nicotinic receptor and inhibition by snake venom toxins. *Neuron* **106**, 952-962 (2020).
6. N. Unwin, Protein-lipid architecture of a cholinergic postsynaptic membrane. *IUCrJ* **7**, 852-859 (2020).
7. G. F. Schröder, A.T. Brunger, M. Levitt, Combining efficient conformational sampling with a deformable elastic network model facilitates structure refinement at low resolution. *Structure* **15**, 1630-1641 (2007).
8. J.J. Fernández, D. Luque, J.R. Castón, J.L. Carrascosa, Sharpening high resolution information in single particle electron cryomicroscopy. *J. Struct. Biol.* **164**, 170–175 (2008).
9. C. Toyoshima, N. Unwin, Ion channel of acetylcholine receptor reconstructed from images of postsynaptic membranes. *Nature* **336**, 247-250 (1988).
10. B. Zuber, N. Unwin, The structure and superorganisation of acetylcholine receptor-rapsyn complexes. *Proc. Natl. Acad. Sci. USA* **110**, 10622-10627 (2013).
